# Still a filtering failure? A follow-up commentary on automated MeSH indexing

**DOI:** 10.5195/jmla.2026.2377

**Published:** 2026-10-01

**Authors:** Nicole Askin, Tyler Ostapyk, Carla Epp

**Affiliations:** 1 nicole.askin@umanitoba.ca, Liaison Librarian, Winnipeg Regional Health Authority Virtual Library, University of Manitoba, Winnipeg, MB, Canada; 2 tyler.ostapyk@umanitoba.ca, Liaison Librarian, Winnipeg Regional Health Authority Virtual Library, University of Manitoba, Winnipeg, MB, Canada; 3 carla.epp@umanitoba.ca, Liaison Librarian, Winnipeg Regional Health Authority Virtual Library, University of Manitoba, Winnipeg, MB, Canada

**Keywords:** Automated MeSH indexing, filter, commentary

## Abstract

We previously found that automated indexing in PubMed and Medline increased the risk of inappropriately excluding human studies when using the human studies filter from the Cochrane Highly Sensitive Search Strategy (HSSS). Following the National Library of Medicine's transition from the Medical Text Indexer-Auto (MTIA) to the Medical Text Indexer-Next Generation (MTIX) in 2024, we wanted to assess whether that was still the case. Using the same approach as our previous study, we reviewed a set of MTIX-indexed records that were excluded by the HSSS. Only 1.1% turned out to be human studies that were inappropriately excluded – a major improvement over not only MTIA but also human indexers in our previous comparison. However, problems remain with automated indexing and search filters. This commentary outlines our findings and our ongoing concerns.

## INTRODUCTION

Medical Subject Headings (MeSH) are a key controlled vocabulary for health librarians. They are used extensively in knowledge synthesis searching both to discover relevant records and to exclude those which are irrelevant, as exemplified by the Cochrane Highly Sensitive Search Strategies (HSSS), which use the string ‘exp animals/ not humans.sh’ combined with NOT against the rest of the search to limit to human studies (“NOT-NOT” logic) [1]. Traditionally MeSH have been applied to records in PubMed/Medline by human indexers; however, more recently the National Library of Medicine (NLM) has moved to automatically applying algorithmically determined headings to its records. The current algorithm in use, Medical Text Indexer-NeXt Generation (MTIX), uses a machine-learning model for MeSH term assignment. It replaced the previous Medical Text Indexer-Auto (MTIA) algorithm in April 2024 [2].

We previously evaluated the impact of MTIA on inappropriate exclusion of human records through use of the Cochrane HSSS [3]. At that time, we concluded that the HSSS would inappropriately exclude human studies automatically indexed via MTIA at nearly seven times the rate of those indexed by human indexers. We sought to update our previous study to evaluate the implementation of MTIX, to see if our conclusions would change.

## REPEATING OUR METHODS

As reported at CHLA 2025 [4], we sought to assess the potential impact of MTIX on the assignment of human indexing terms and the resulting inappropriate exclusion of human studies from knowledge synthesis searches. To accomplish this, we adopted the methodology of our previous study [3]: we first conducted a search in Ovid Medline using the Cochrane HSSS [1] as our search strategy. We then limited our results to those which were specifically excluded by the non-human-studies string in the period since the implementation of MTIX and which used an automated indexing method.

1. randomized controlled trial.pt.
2. controlled clinical trial.pt.
3. randomized.ab.
4. placebo.ab.
5. clinical trials as topic.sh.
6. randomly.ab.
7. trial.ti.
8. 1 or 2 or 3 or 4 or 5 or 6 or 7
9. exp animals/ not humans.sh.
10. 8 not 9
11. 8 not 10
12. medline.st.
13. 11 and 12
14. and (202405* or 202406* or 202407* or 202408* or 202409* or 202410* or 202411* or 202412*).da.
15. and automated*.ig.

A total of 2,285 unique results were imported into Covidence for screening. The results were screened independently by two team members at the title-abstract and full-text stages. Records were included if they described a human study, according to the definition adopted in our previous paper: (a) the study was interventional and the intervention was performed on a human or human product (human cells, tissues, etc.); (b) the study was comparative and involved a comparison between groups of humans; or (c) a significant outcome of interest was focused on humans. Twenty-five studies meeting this definition were ultimately included and were analyzed using the custom data extraction form developed for our previous study.

As part of the extraction process, we considered potential reasons why papers might be misidentified as non-human studies. The most frequent cause was a single record which described multiple studies, at least one of which was a human study – for example, combining an observational study in humans with an experimental study on animal subjects, which is then indexed only using animal terms. This cause is consistent with the performance of both MTIA and human indexers in our previous paper.

Our previous study found that 4.2% of articles excluded by the non-human-studies string and indexed via MTIA were in fact human studies, compared to 2.2% in the set of studies indexed by human indexers. In this update, 25 of 2,285 studies screened, or 1.1%, were inappropriately excluded human studies. Thus, according to our analysis, MTIX outperformed not only MTIA but also the human indexers in our previous study.

This is consistent with expectations from NLM. As they reported in their launch of MTIX, they found that F1 scores (combining recall and precision) in MTIX-indexed records were 96% for the MeSH term “humans” and 92% for “animals” [5]. However, terms that were used less consistently by human indexers (which was the indexing method for the algorithm’s training set) perform less well [6].

## SO, WE’RE ALL GOOD NOW?

At first glance, our repeated study has a positive outcome, and we do not wish to downplay the benefit of having better indexing moving forward. However, there continues to be a major stumbling block in using this filter for knowledge synthesis: while some individual records have been curated by human indexers – a few even before our previous study was published – others remain uncorrected even years after publication and continue to be inappropriately excluded by the filter. Moreover, hundreds of knowledge synthesis projects published while MTIA indexing was in effect used the Cochrane Highly Sensitive Search Strategy.^*^ It is unknown what impact potentially missing human studies may have had on their findings.

Although curation addressed the misindexing of some of the records in our study, this practice is limited: as reported on the expertsearching listserv, curation is targeted only to high-impact publication types and population groups, does not generally add missing headings outside a narrow set, and “curators are instructed to leave terms in place unless they are sure they are wrong” [7]. NLM has reported that they are considering using MTIX to update old records, but that such a project would be limited in scope [2]. As noted by Amar-Zifkin and colleagues, it would also impact replicability of prior searches [8].

## UH OH

There is also evidence to suggest that the problem extends beyond human studies filtering to encompass other purely MeSH-based approaches to study inclusion or exclusion. For example, a MeSH string is often used to limit results by age group or by sex/gender. As one author presented at CHLA 2025 [9], there was a sharp decline in use of major age-related MeSH terms like “adult” and “newborn”, as well as “female” and “male”, in Medline records during the period in which MTIA was in use. This is consistent with the pilot study by Chen and colleagues using the Interactive MTI tool [10]. While to our knowledge no study has yet evaluated the practical effect of these indexing deficiencies, it seems very likely that they would impact MeSH-based filtering of records by age or sex/gender.

Other authors have described further problems with MTIA indexing which may impact discoverability of records. For example, Amar-Zifkin and colleagues found cases of both inappropriately assigned and inappropriately omitted MeSH terms [11]. Philippopoulos found that no MTIA-indexed records included the MeSH for “intersex persons”, despite the presence of around 100 records that should have included it [12]. Wilson found that MTIA often misunderstood figurative language in applying the MeSH term “Malus” (apple tree) to records in which the term “apple” was used metaphorically [13]; while this example resulted in overbroad rather than overspecific search results, it suggests the possibility of studies using more figurative terms being missed due to literal interpretation. Again, it is unclear what impact these have had on already published reviews, or ongoing or future reviews that will search records from this period.

It is also possible that problems with MeSH indexing may impact searches in other databases. For example, Embase uses MeSH terms for “articles from other MEDLINE titles (with a focus on basic biomedicine, Allied Health and other topics that are peripheral to the core topics of Embase)” [14]. Searches on these topics in Embase would thus also be affected by problems with MeSH indexing, particularly given that these are areas that already tend towards ambiguous terminology, as explored by Amar-Zifkin and colleagues [8]. Additionally, while EBSCO reports only using human indexers [15], our informal investigation of indexing problems has led to the incidental finding that the Human heading is often absent from CINAHL records of human studies, despite the records being otherwise well-indexed. It is unclear why this is the case, but it does suggest that limiting to human studies in CINAHL could be of questionable validity for knowledge synthesis searching.

## WHAT NOW?

MTIX represents a significant improvement over MTIA in terms of accuracy of human/animal indexing. However, records which were indexed by MTIA still exist, and are still potentially relevant to knowledge synthesis searches. Therefore, we echo our previous conclusions: we urge information specialists conducting knowledge synthesis projects in Medline or PubMed to exercise caution in using pure-MeSH filters for searches that will encompass the period in which MTIA was in use. In fact, given the findings of colleagues, we would extend that caution to any use of exclusively MeSH-based approaches for records from this period.

It is also advisable to consider other methods to ensuring the discoverability of research, such as through author-supplied keywords or an amended approach to abstract writing. This is particularly important given that MTIX does not have access to the full text of the article, whereas human indexers did [6]. Allen and colleagues have proposed an author checklist to mitigate indexing problems [16].

Indexing concerns in databases other than PubMed/Medline are relatively understudied. There have been some studies of indexing discrepancies in Embase’s Emtree [17], but more work is needed to understand if and how indexing algorithms and practices may impact searches in other databases.

## Footnotes

* While inexact, a search of Google Scholar for (“exp animals not” intitle:review) limited to publications between 2021 and 2024 returns 367 results.
